# Development of a new minimally invasive phototherapy for lung cancer using antibody–toxin conjugate

**DOI:** 10.1111/1759-7714.14776

**Published:** 2023-01-19

**Authors:** Takumi Sonokawa, Naoko Obi, Jitsuo Usuda, Yukio Sudo, Takao Hamakubo

**Affiliations:** ^1^ Department of Thoracic Surgery Nippon Medical School Tokyo Japan; ^2^ Research & Development Division PhotoQ3 Inc. Tokyo Japan

**Keywords:** drug delivery system, endosomal escape, immunotoxin, mono‐l‐aspartyl chlorin e6 (NPe6), photodynamic therapy

## Abstract

**Background:**

Photodynamic therapy (PDT) is a cancer‐targeted treatment that uses a photosensitizer (PS) and laser irradiation. The effectiveness of current PDT using red light for advanced cancers is limited, because red light can only reach depths within a few millimeters. To enhance the antitumor effect for lung cancers, we developed a new phototherapy, intelligent targeted antibody phototherapy (iTAP). This treatment uses a combination of immunotoxin and a PS, mono‐L‐aspartyl chlorin e6 (NPe6).

**Methods:**

We examined whether cetuximab encapsulated in endosomes was released into the cytosol by PS in PDT under light irradiation. A431 cells were treated with fluorescein isothiocyanate‐labeled cetuximab, NPe6, and light irradiation and were observed with fluorescence microscopy. We analyzed the cytotoxicity of saporin‐conjugated cetuximab (IT‐cetuximab) in A431, A549, and MCF7 cells and the antitumor effect in model A549‐bearing mice in vivo using the iTAP method.

**Results:**

Fluorescent microscopy analysis showed that the photodynamic effect of NPe6 (20 μM) and light irradiation (37.6 J/cm^2^) caused the release of cetuximab from the endosome into the cytosol. In vitro analysis demonstrated that the iTAP method enhanced the cytotoxicity of IT‐cetuximab by the photodynamic effect. In in vivo experiments, compared with IT‐cetuximab alone or PDT alone, the iTAP method using a low dose of IT‐cetuximab showed the greatest enhancement of the antitumor effect.

**Conclusions:**

Our study is the first report of the iTAP method using NPe6 for lung cancer cells. The iTAP method may become a new, minimally invasive treatment superior to current PDT methods.

## Graphical abstract

The combination of saporin‐conjugated anti‐epidermal growth factor receptor (EGFR) antibody as an immunotoxin and NPe6 with light irradiation had enhanced antitumor effect. We named this therapeutic method as intelligent targeted antibody phototherapy (iTAP). This study indicates that iTAP showed a stronger antitumor effect than conventional phototherapy or immunotoxin only. This new phototherapy can provide a minimally invasive treatment method for elderly lung cancer patients, and iTAP may be applicable to other solid cancers.

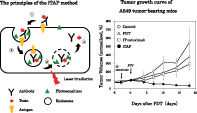

## INTRODUCTION

Photodynamic therapy (PDT) is a cancer‐targeted treatment that uses a tumor‐affinity photosensitizer (PS) and low‐power laser irradiation.^1^, ^2^, ^3^, ^4^, ^5^ The strong oxidative effect of singlet oxygen and reactive oxygen species (ROS) generated by the photochemical reaction between laser light and PS causes damage to tumor cells. Unlike conventional therapies such as surgery, PDT is minimally invasive and can be performed repeatedly with no cumulative toxicity.^5^, ^6^ PDT is widely used to treat various cancers. In the field of lung cancer, PDT is established as one of the standard treatment options for centrally located early lung cancers and is also used as a palliative treatment for centrally located advanced lung cancers.^2^, ^4^, ^5^, ^6^, ^7^

The depth of light penetration into the tissue depends on the wavelength of the light. Currently, mono‐L‐aspartyl chlorin e6 (NPe6, talaporfin sodium, Laserphyrin; approved in Japan for PDT) is used as a second‐generation PS. NPe6 has a major absorption band at 664 nm, which is longer than that of the porfimer sodium previously used for PDT and therefore, can reach deeper regions.^1^, ^2^, ^3^ Recently, indications for NPe6‐PDT have been expanded to include malignant brain tumors and esophageal cancer.^8^, ^9^ However, even red light can only reach depths within a few millimeters of the tissue surface.^5^, ^10^, ^11^ Therefore, the effectiveness of PDT for cancers located deep in the body is limited. New technological breakthroughs are needed for PDT to achieve satisfactory results for deeper and larger tumors.

Recently, the photochemical internalization (PCI) technique has been introduced to induce the effective uptake of anticancer drugs. PCI is a technique that uses biphasic PS, which tends to localize to the cell membrane, to release molecules encapsulated in endosomes into the cytosol in response to light irradiation.^12^, ^13^, ^14^ When PS is excited by light irradiation, the membranes of endosomes and lysosomes are disrupted by the generation of ROS, predominantly singlet oxygen, and encapsulated molecules are released into the cytosol. Singlet oxygen has a short half‐life (~0.01–0.04 μs) (i.e., its effects are localized).^15^ Therefore, PCI aims to reduce drug side effects and achieve the desired effect within a smaller dose.^13^

We previously reported that saporin‐conjugated anti‐Robo1 (one of the receptors involved in cancer progression) antibody, an immunotoxin, enhances cytotoxic effects in combination with the PS, disulfonated aluminum phthalocyanine (AlPcS_2a_), and light irradiation.^16^, ^17^ The aim of this study was to enhance the antitumor effect of the immunotoxin using NPe6, a dye used for PDT, as a photosensitizer and laser irradiation. We named this therapeutic method as intelligent targeted antibody phototherapy (iTAP). The iTAP is a novel treatment method that has never been reported before. In this study, we used an immunotoxin in which saporin was conjugated to cetuximab, an anti‐epidermal growth factor receptor (EGFR) antibody. Saporin is a toxic protein with a molecular weight of ~30 kDa and is classified as a type 1 ribosome‐inactivating protein (RIP) isolated from the plant *Saponaria officinalis*. This molecule does not have a natural cell‐binding domain and is active only when transferred to the cytosol after endocytosis.^18^, ^19^ The antitumor principle of the iTAP method involves a high concentration of saporin delivered to cancer cells, specifically by cetuximab, which accumulates in endosomes. Subsequently, on laser irradiation of NPe6, the photodynamic effect causes saporin to translocate from the endosome to the cytosol, leading to cell death. Accordingly, a strong antitumor effect was expected. The combination of light therapy using immunotoxin and PS is expected to improve the antitumor effect and expand the potential of phototherapy in cancer treatment.

## MATERIAL AND METHODS

### Cell lines

The A431, A549, and MCF7 cell lines were obtained from KAC. All the cells were cultured in a humidified incubator at 37°C with 5% CO_2_ and 70% relative humidity. A431 and A549 cells were grown in high‐glucose Dulbecco's modified Eagle's medium with 10% fetal bovine serum (FBS). MCF7 cells were grown in Eagle's minimum essential medium supplemented with non‐essential amino acids and 10% FBS.

### Immunotoxin

In this study, we used an immunotoxin in which saporin was conjugated to cetuximab (Selleck.co.jp), hereafter referred to as IT‐cetuximab. IT‐cetuximab was prepared as follows: biotinylated cetuximab was purified by mixing cetuximab with EZ‐LINK sulfo‐NHS‐LC‐biotinylation kit (Thermo Fisher Scientific) at a 1:40 molar ratio using PD SpinTrap G‐25 (GE Healthcare Life Sciences). Next, biotinylated cetuximab and streptavidin‐saporin (Biotin‐Z Internalization Kit [KIT‐27‐Z]) (Advanced Targeting Systems) were mixed in equivalent amounts and allowed to react at room temperature for 30 min to obtain IT‐cetuximab.

### Photosensitizer

Mono‐L‐aspartyl chlorin e6 (NPe6, talaporfin sodium, Laserphyrin) was purchased from Meiji Seika Pharma. NPe6 is a water‐soluble photosensitizer approved for PDT, with a maximum absorption peak at 407 nm and a second peak at 664 nm.^4^, ^20^

### Light sources

A light‐emitting diode (LED) lamp (54 W) with a peak wavelength of 650 nm was purchased from King Do Way (18PCS E27) (Amazon.co.jp). An LED lamp was used in the cytotoxicity experiments.

A diode laser (Meiji Seika Pharma) emitting a laser light with a wavelength of 664 nm was used as the light source. This wavelength matches the absorption bands of NPe6. This laser unit was licensed for PDT using NPe6.

### Fluorescence microscopy analysis

Cetuximab was labeled with a fluorescein isothiocyanate (FITC)‐streptavidin conjugate. The FITC‐streptavidin conjugate was purchased from Tokyo Chemical Industry. The FITC‐cetuximab conjugate was prepared by mixing equal amounts of biotinylated cetuximab and FITC‐streptavidin and incubating at room temperature for 30 min.

Because FITC has been reported to increase optical density as pH increases,^21^ the fluorescence intensity increases when FITC is released into the cytosol.^22^ Therefore, to visualize the endosomal escape because of photodynamic effect, we evaluated the fluorescence intensity of FITC.

A431 cells were seeded on 35 mm glass‐based dishes (AGC Techno Glass) at a density of 3 × 10^5^ cells per dish. Two days after incubation, the cultured cells were treated in the following four ways: (1) cetuximab‐FITC, (2) cetuximab‐FITC + light irradiation, (3) cetuximab‐FITC + NPe6, and (4) cetuximab‐FITC + NPe6 + light irradiation. The doses of cetuximab‐FITC and NPe6 were 1 nM and 20 μM, respectively. Twenty‐one hours after drug administration, cells in groups 2 and 4 were irradiated with LED light for 10 min (37.6 J/cm^2^). The cells were then observed using a confocal microscope (Fluo View FV1000; Olympus). The fluorescence images were analyzed using ImageJ software (National Institutes of Health) to quantitatively determine the mean fluorescent intensity (MFI), the average of the difference between the fluorescence intensity of the three intracellular areas and that of the extracellular area.

### Flow cytometry

The amount of the EGFR antigen on the cell surface (antigen molecules/cell) was quantified from the histogram of calibration beads of QIFIKIT (Agilent Tech).

Overall, 1 × 10^5^ A431, A549, and MCF7 cells were incubated for 1 h at 4°C with 10 μg/mL of the anti‐EGFR monoclonal antibody (Abcam plc) or the isotype control mIgG2a antibody (R&D Systems) in sorting buffer (phosphate buffered saline [PBS] containing 1% bovine serum albumin, 0.1 mM ethylenediaminetetraacetic acid, and 0.1% ProClin 300). After washing thrice with the sorting buffer, cells were incubated for 1 h at 4°C with FITC‐conjugated anti‐mouse IgG from QIFIKIT; standard beads coated with a known amount of mouse IgG molecules also were labeled with this secondary antibody. The labeled samples were washed thrice with the sorting buffer and analyzed using Guava easyCyte Plus Flow Cytometer (Merck). The number of antibody binding sites per cell was calculated by comparing the MFI value of the labeled cells with a calibration curve obtained by regression analysis of the MFI values of the standard beads.

### Immunotoxin cytotoxicity assay

A431, A549, and MCF7 cells were seeded at 10 × 10,^3^ 2.5 × 10,^3^ and 7.5 × 10^3^ cells per well, respectively, in 96‐well plates, cultured for 24 h, and then exposed to various concentrations (0.001–8 nM) of IT‐cetuximab and/or NPe6 (30 μM for A431 and A549, and 20 μM for MCF7). Twenty‐four hours after administration of these drugs, cells were washed by 100 μl/well PBS and irradiated from the LED lamp for 10 min (37.6 J/cm^2^), and cell viability was determined 48 h after irradiation using a cell counting kit‐8 (Dojindo Laboratories), according to the instruction manual. The half‐maximal inhibitory concentrations (IC_50_) were evaluated from the sigmoid curve obtained using the curve‐fitting tool of the ImageJ software.

### Animal and tumor models

Female BALB/c nude mice (5‐week‐old) were obtained from Japan SLC. All mice were housed and maintained under optimal light, temperature, and humidity conditions, with free access to food and water in the animal facility at Nippon Medical School.

### In vivo treatment protocols

BALB/c nude mice were inoculated subcutaneously in the right hind flank with 1 × 10^7^ A549 cells in a volume of 0.1 mL. Treatment was initiated when the tumor reached 6–7 mm in diameter.

#### Pre‐experiments to determine the dose of IT‐cetuximab

First, we determined the appropriate dose of IT‐cetuximab. Mice were intraperitoneally injected with IT‐cetuximab at doses of 0 (control), 0.1, 1, and 3 mg/kg (*n* = 3). Tumor size was determined using caliper measurements, and tumor volumes were calculated using the following formula: $\text{tumor volume}=\frac{L\times D^{2}}{2}$, where *L* is the long diameter, and *D* is the short diameter. The tumor volume was normalized based on the value at day 0.

#### In vivo treatment protocols using antibodies

The mice were randomized into four groups as follows: (1) control, (2) IT‐cetuximab, (3) NPe6 + light irradiation (PDT), and (4) IT‐cetuximab and PDT (iTAP) (*n* = 3). In the PDT arm, 2 h after the administration of NPe6 (5 mg/kg) via the tail vein, the tumors were irradiated with a 664 nm laser at a dose of 30 J/cm^2^ from the diode laser unit. In the iTAP arm, mice were injected intraperitoneally with 3 mg/kg of IT‐cetuximab, and 2 days later, PDT was performed as in the PDT arm. The antitumor effect was evaluated by measuring the tumor volume. Tumor size was determined using caliper measurements, and tumor volumes were calculated as described vide supra.

### Ethics

All animal experiments were conducted according to protocols approved by the Animal Care and Use Committee of Nippon Medical School (approval number: 2020‐095).

### Statistical analysis

Data are shown as the mean ± standard deviation. Statistical analysis was performed using IBM Statistical Package for Social Sciences (SPSS) software (version 27). Comparisons between groups were performed using analysis of variance with post‐hoc Tukey test. Statistical significance was set at *p* < 0.05.

## RESULTS

### Cetuximab is released from endosomes by the photodynamic effect using NPe6 and light irradiation

To elucidate the mechanism of iTAP, we examined whether cetuximab in the endosome was released into the cytosol via the photodynamic effect of NPe6 on light irradiation. A431 cells were treated with FITC‐labeled cetuximab and NPe6, light irradiation, or NPe6 plus light irradiation.

FITC has been reported to increase optical density as pH increases.^21^ When FITC is translocated from the endosome into the cytosol by the photodynamic effect, the fluorescence intensity increases because of its pH.^22^

**FIGURE 1:**
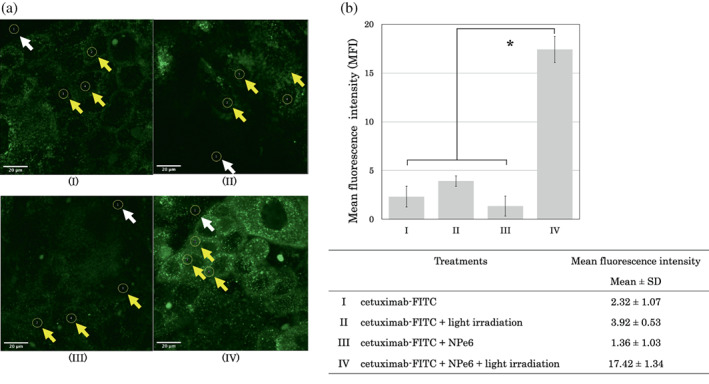
Fluorescence microscopic analysis of cetuximab release into the cytosol by the photodynamic effect. (a) Fluorescence microscopy images of A431 cells treated in four different ways: (I) cetuximab‐fluorescein isothiocyanate (FITC), (II) cetuximab‐FITC + light irradiation, (III) cetuximab‐FITC + NPe6, and (IV) cetuximab‐FITC + NPe6 + light irradiation. (b) Mean fluorescence intensity (MFI) in the cells was quantitatively determined using ImageJ and is presented graphically. MFI was calculated as the average of the difference between the fluorescence intensity of the three intracellular areas (yellow arrows) and that of the extracellular area (white arrows). The fluorescence intensity values for each treatment are listed. The values of IV were significantly higher than that of the other conditions (AVOVA, *p < 0.05). Data are shown as the mean ± standard deviation.

Figure 1(a) shows the fluorescence microscopic views of FITC‐labeled cetuximab to estimate its location in the cell. Fluorescence intensity was quantitatively determined, and the fluorescence intensities of cetuximab‐FITC, cetuximab‐FITC + light irradiation, cetuximab‐FITC + NPe6, and cetuximab‐FITC + NPe6 + light irradiation were 2.32 ± 1.07, 3.92 ± 0.53, 1.36 ± 1.03, and 17.42 ± 1.34, respectively (mean ± SD) (Figure 1(b)). The fluorescence intensity of IT‐cetuximab + NPe6 + light irradiation was significantly higher than that of other conditions (ANOVA, *p* < 0.05).

These results indicated that the photodynamic effect of NPe6 (20 μM) and light irradiation (37.6 J/cm^2^) caused the release of cetuximab from the endosome into the cytosol. We hypothesized that saporin‐conjugated cetuximab would be released into the cytosol in iTAP, inducing saporin cytotoxicity.

### Cytotoxic effect of immunotoxin using the principles of the iTAP method

**FIGURE 2:**
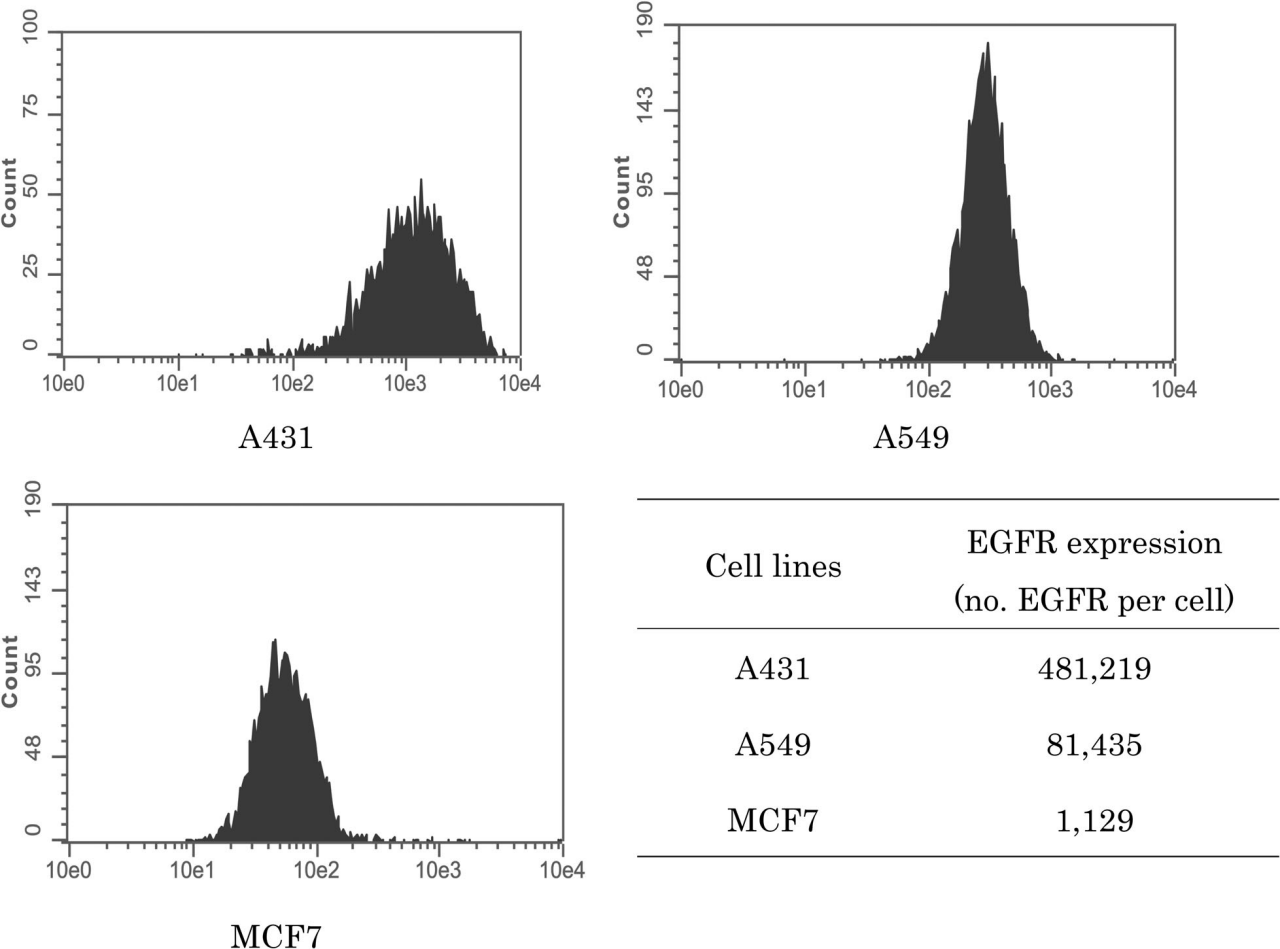
The expression level of epidermal growth factor receptor (EGFR) in each of the cell lines. Analysis of EGFR expression level on the cell surface was performed by flow cytometry using QIFIKIT. Histograms showed staining of A431, A549, and MCF7 cells.

We analyzed the cytotoxicity of IT‐cetuximab (saporin conjugated with cetuximab) in several cell lines using the iTAP method. First, we examined the EGFR expression level on the cell surface by flow cytometric analysis. The EGFR number on the cell surface of each cell line was 481 219/cell, 81 435/cell, and 1129/cell, for A431, A549, and MCF7, respectively (Figure 2).

**FIGURE 3:**
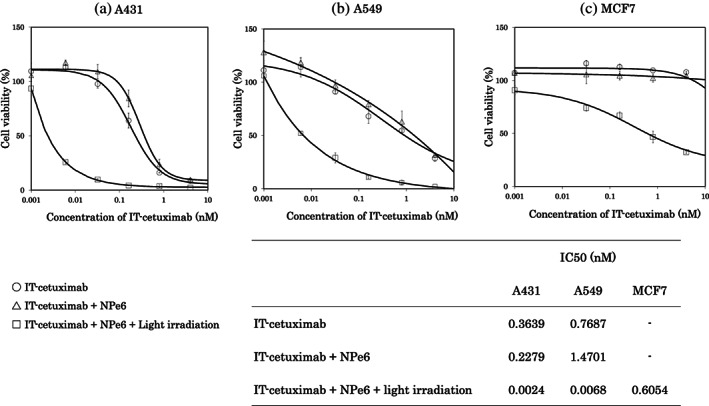
Cell viability assay for antitumor effect. We analyzed the cytotoxicity of IT‐cetuximab combined with NPe6‐PDT in A431 (a), A549 (b), and MCF7 (c) cells, and evaluated the half‐maximal inhibitory concentration (IC_50_) of each cell line from the sigmoid curve. The cytotoxicity of IT‐cetuximab was enhanced by the combination of NPe6‐PDT. This effect was even observed in MCF7 cells with low EGFR expression (c). Data are shown as the mean ± standard deviation. IT‐cetuximab, saporin‐conjugated cetuximab; PDT, photodynamic therapy; EGFR, epidermal growth factor receptor

**FIGURE 4:**
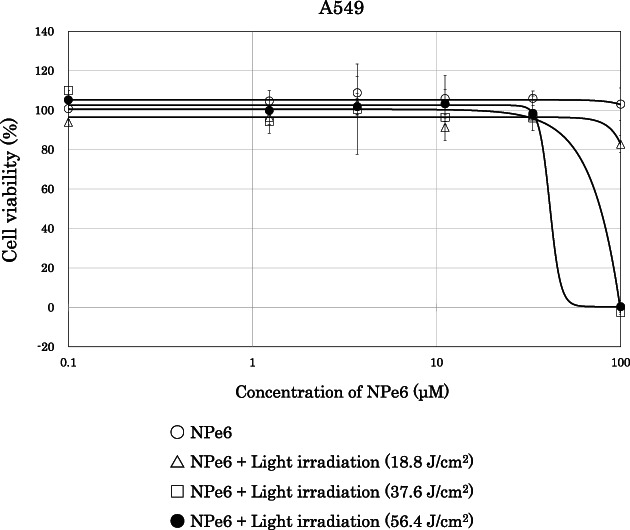
Cell viability assay of NPe6‐PDT for A549. We examined the cytotoxic effect of PDT with different concentrations of NPe6 and amounts of light irradiation. A549 cells were seeded at 2.5 × 10^3^ cells per well in 96‐well plates, cultured for 24 h, and then exposed to various concentrations (1–100 μM) of NPe6. After 21 h, cells were washed with 100 μl/well of PBS, and 3 h later, cells were irradiated with LED light for 5, 10, and 15 min (18.8, 37.6, and 56.4 J/cm^2^). Cell viability was determined 48 h after LED irradiation using cell counting kit‐8. The dose of PDT in the experiments shown in Figure 3(b) (30 μM NPe6 and 37.6 J/cm^2^ light irradiation) did not exert cytotoxicity by itself. PDT, photodynamic therapy; PBS, phosphate buffered saline; LED, light‐emitting diode

Next, we examined the inhibitory effect of IT‐cetuximab on the growth of A431 cells, in which EGFR was highly expressed.^23^ The cytotoxicity of cetuximab increased in a dose‐dependent manner (Figure 3(a)). The cytotoxicity of IT‐cetuximab plus NPe6 was comparable to that of IT‐cetuximab alone. However, the cytotoxicity was significantly enhanced when IT‐cetuximab was combined with NPe6 and low dose PDT light irradiation (30 μM NPe6 and LED light at 37.6 J/cm^2^ for 10 min). The enhanced cytotoxic effect was conspicuous, even at lower doses of IT‐cetuximab. The IC_50_ values of IT‐cetuximab, IT‐cetuximab + NPe6, and IT‐cetuximab + NPe6 + irradiation (iTAP) evaluated from the cell viability sigmoidal curve were 0.3639 nM, 0.2279 nM, and 0.0024 nM, respectively (Figure 3). The results showed that the new treatment, iTAP (saporin‐conjugated cetuximab plus low dose PDT) had a strong cytotoxic effect against cancer cells. As shown in Figure 3(b), iTAP caused a strong cytotoxic effect in A549 cells with a K‐ras mutation. Incidentally, as shown in Figure 4, the dose of PDT (30 μM NPe6 and 37.6 J/cm^2^ light irradiation) did not exert cytotoxicity by itself. The IC_50_ of IT‐cetuximab, IT‐cetuximab + NPe6, IT‐cetuximab + NPe6 + irradiation (iTAP) was 0.7687 nM, 1.4701 nM, 0.0068 nM, respectively.

A cytotoxicity assay using a similar protocol conducted on MCF7, a breast cancer cell line known for low EGFR expression,^24^, ^25^ showed enhanced cytotoxicity with the combination of NPe6 and light irradiation (Figure 3(c)). The IC_50_ of IT‐cetuximab + NPe6 + irradiation (iTAP) was 0.6054 nM. These results indicate that this new treatment, iTAP, can cause a strong cytotoxic effect even in cancer cells with low EGFR expression.

### iTAP causes a strong antitumor effect against A549 tumors in an in vivo model

**FIGURE 5:**
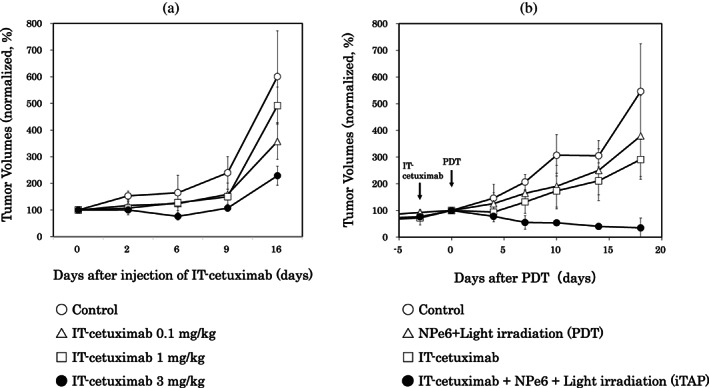
Tumor growth curve of A549 tumor‐bearing mice after different treatments. (a) Pre‐experiments to determine the dose of IT‐cetuximab. Mice were injected intraperitoneally with IT‐cetuximab at several doses. Injection of IT‐cetuximab at 3 mg/kg significantly inhibited tumor growth compared to control (ANOVA, *p* < 0.01), but the tumors did not achieve complete response and showed a tendency for regrowth from the 6th day after injection. (b) In vivo tumor growth in mice treated with the following: (1) control, (2) IT‐cetuximab, (3) NPe6 + light irradiation (PDT), and (4) IT‐cetuximab and PDT (iTAP) (*n* = 3). Compared to controls, IT‐cetuximab alone or PDT alone did not show much enhancement of antitumor effect, but the iTAP method using low dose of IT‐cetuximab and NPe6 with laser irradiation showed the most enhanced antitumor effect (ANOVA, *p* < 0.01). The tumor volume is normalized based on the day 0 value. Data are shown as the mean ± standard deviation. PDT, photodynamic therapy; IT‐cetuximab, saporin‐conjugated cetuximab; iTAP, intelligent targeted antibody phototherapy

To determine the appropriate dose of cetuximab for iTAP, we conducted in vivo experiments. We selected the human lung cancer cell line A549 as a tumor model for in vivo experiments. Mice were intraperitoneally injected with various concentrations of IT‐cetuximab (0, 0.1, 1, and 3 mg/kg). Injection of IT‐cetuximab at 3 mg/kg inhibited tumor growth compared to the control (ANOVA, *p* < 0.01); however, the tumor showed a regrowth 6 days after the injection of IT‐cetuximab (Figure 5(a)). This result indicated that IT‐cetuximab alone, even at 3 mg/kg, did not completely inhibit tumor growth. Therefore, we determined that 3 mg/kg was the optimum dose for iTAP (Figure 5(a)).

We investigated the iTAP method by randomly assigning mice to four treatment groups, as described in *In vivo treatment protocols using antibodies*. Figure 5(b) shows the progress of the tumor volume. Compared to controls, IT‐cetuximab (3 mg/kg) alone or PDT (5 mg/kg NPe6 and 30 J/cm^2^ laser irradiation) alone did not show a greatly enhanced antitumor effect at this dose. However, the iTAP method using a low dose of IT‐cetuximab and NPe6 with laser irradiation showed the greatest enhancement of the antitumor effect (ANOVA, *p* < 0.01). In the iTAP arm, tumor recurrence was completely suppressed. This result showed that the iTAP method, combining low doses of IT and PDT, strongly induces cell death by saporin toxicity.

## DISCUSSION

**FIGURE 6:**
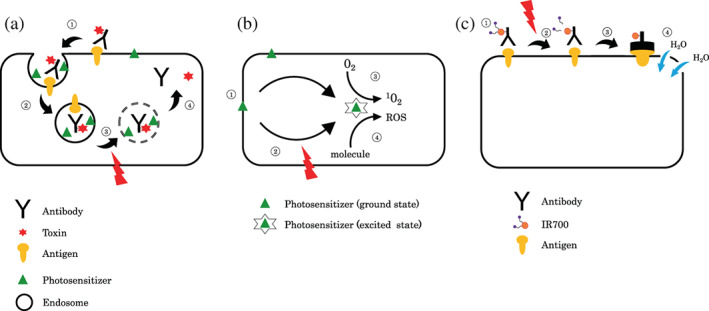
Schema of the action of mechanisms and comparison of three methods of phototherapy. (a) iTAP method, (b) PDT, and (c) PIT. Each of these methods requires multiple steps to achieve its antitumor effect. (a) Schema of the principles of the iTAP method. (1) Binding of antibodies to antigens expressed on the cell surface. (2) Endocytosis. (3) Endosome permeability increased by light irradiation. (4) Toxin translocation into the cytoplasm and cell death. (b) Schematic of the mechanism of action of PDT. (1) Accumulation of the PS to tumor cells. (2) On laser irradiation, the PS is transferred from its ground state to an excited state. (3) The excited‐state PS transfers energy to ground‐state oxygen, generating singlet oxygen. (4) The excited‐state PS reacts with a neighboring molecule, producing various ROS. (c) Schematic of the mechanism of action of PIT. (1) Binding of IR700‐conjugated antibodies to antigens expressed on the cell surface. (2) Red light irradiation releases a portion of IR700 from the antibody‐IR700 conjugate. (3) Physical changes in the antibody‐IR700 conjugate. (4) Physical stress on the local cell membrane and cell rupture. iTAP, intelligent targeted antibody phototherapy; PDT, photodynamic therapy; PIT, photoimmunotherapy; PS, photosensitizer; ROS, reactive oxygen species

In this study, we found that the combination of immunotoxins and PDT using NPe6 enhanced the antitumor effect. The antitumor effect of the iTAP method should be mainly because of the cytotoxicity of saporin released into the cytosol by endosomal escape. In this approach, saporin accumulated at high concentrations in cancer cells, using the tumor specificity of EGFR, and saporin exerted its enzymatic activity only in light‐irradiated cells (Figure 6(a)).

The main mechanism of the antitumor effect of PDT is the strong oxidative effect of singlet oxygen and ROS generated by the photochemical reaction between laser light and PS (Figure 6(b)).^1^, ^2^, ^3^, ^4^, ^5^, ^6^ In addition to these direct cytotoxic effects, PDT also causes microvascular damage around tumors and elicits antitumor effects through various immune responses.^1^, ^5^, ^26^, ^27^ PDT achieves good results for early‐stage cancers, but has limited efficacy for advanced cancers.^11^

The main cytotoxic effect of NPe6 is considered to be related to lysosomal enzyme release and the activation of mitochondrial Bcl2‐mediated apotosis.^28^, ^29^ Moreover, several reports have been published on the mechanism of cellular uptake of NPe6. It has been reported that NPe6 may be taken up into the cell by the endosomal pathway as well as translocation to the cell membrane.^30^, ^31^ Recently, Saito et al.^32^ revealed that NPe6 is taken in by endocytosis and translocates from early endosomes to lysosomes in cancer cell lines. In this study, we showed that NPe6 and light irradiation induced endosomal escape of immunotoxins (Figure 1). These results suggest that NPe6 may coexist with immunotoxins through the mechanism of endocytotic uptake, assist the endosomal escape of immunotoxin by the photodynamic effect, and induce strong saporin cytotoxicity (Figure 6(a)). The amount of light irradiation sufficient to induce endosomal escape by the photodynamic effect with NPe6 was not cytotoxic by itself (Figure 4). The strong antitumor effect by iTAP shown in Figure 5(b) suggests the synergistic effect of PDT and endosomal escape of immunotoxin.

PDT is not indicated for large tumors because the therapeutic effect is insufficient if the light does not penetrate the tissue sufficiently.^11^, ^33^ The amount of light irradiation and the depth to which the light reaches are important factors that determine the antitumor effect of PDT. We found that the iTAP method had a strong antitumor effect with a small amount of light irradiation as compared with that reported in Usuda et al.^20^, ^34^ The reason iTAP is more necrotic than PDT, although the light wavelengths are the same, is that PDT requires a certain amount of light energy to produce an antitumor effect. Moreover, iTAP can produce a strong antitumor effect even with weak light, as long as the light energy is sufficient to induce endosomal escape of saporin. Therefore, iTAP will be applicable to large tumors, tumors located in areas that are difficult to irradiate with light, and metastatic lung tumors that are not indications for conventional PDT. The iTAP method is a new treatment modality with great potential to expand the arsenal against recalcitrant tumors.

Photoimmunotherapy (PIT) is another treatment that combines antibody drugs with phototherapy.^35^, ^36^, ^37^, ^38^ PIT destroys tumor cells by selectively irradiating cells targeted by IRdye700DX (IR700)‐conjugated antibodies with light. On irradiation with red light, the chemical reaction of the antibody‐IR700 conjugate bound to the target on the cell surface rapidly damages the lipid bilayer on the cell surface (cell rupture) and induces immunogenic cell death by leaking cytoplasmic contents (Figure 6(c)).

As with PIT, there is a concern that the iTAP method may be ineffective against tumors with low expression of target antigens on the tumor cell surface. To confirm the effect of iTAP on EGFR expression levels, several cell lines were compared. Figure 2 shows the expression levels of EGFR as measured by QIFIKIT. As shown cell viability assay in Figure 3, iTAP showed the strongest cytotoxic effect on A431 cells, in which EGFR was highly expressed. However, it also showed a cytotoxic effect on MCF7, in which EGFR was lowly expressed (Figure 3(c)). This means that iTAP is sufficiently effective for heterogeneous tumors in which the EGFR expression level is variable. Because iTAP is a combination therapy with PDT, the antitumor effect of PDT can induce cell death for EGFR‐negative cells for which iTAP is not effective.

Taken together, our results suggest iTAP to have a strong drug effect and may ultimately achieve therapeutic efficacy at lower drug doses and with reduced laser irradiation. Moreover, iTAP is expected to have higher antitumor efficacy than either conventional phototherapy. The iTAP method has a strong antitumor effect and may have the potential to be a new anticancer treatment.

## CONCLUSION

In this study, the iTAP method, which combines toxin‐conjugated antibodies and NPe6 with light irradiation, showed stronger antitumor effects than the conventional PDT. iTAP has the potential to achieve antitumor effects, even in tumors with low expression of targeted antigens on cancer cells.

## AUTHOR CONTRIBUTIONS

All authors had full access to the data in the study and take responsibility for the integrity of the data and the accuracy of the data analysis. Conceptualization: YS and TH. Investigation: TS, NO, JU and TH. Writing – original draft: TS. Writing – review and editing: NO, JU and TH.

## CONFLICT OF INTEREST

The authors have no competing interests to declare.
